# Hepatocellular Carcinoma in Sub-Saharan Africa

**DOI:** 10.1200/GO.20.00425

**Published:** 2021-05-27

**Authors:** V. V. Pavan Kedar Mukthinuthalapati, Vikash Sewram, Ntokozo Ndlovu, Stephen Kimani, Ashraf Omar Abdelaziz, Elizabeth Yu Chiao, Ghassan K. Abou-Alfa

**Affiliations:** ^1^ Memorial Sloan Kettering Cancer Center, New York, NY; ^2^ University of Massachusetts Medical School—Baystate Health, Springfield, MA; ^3^ Department of Global Health, Faculty of Medicine and Health Sciences, African Cancer Institute, Stellenbosch University, Cape Town, South Africa; ^4^ University of Zimbabwe College of Health Sciences, Harare, Zimbabwe; ^5^ The University of North Carolina at Chapel Hill, Chapel Hill, NC; ^6^ Cairo University, Cairo, Egypt; ^7^ MD Anderson Cancer Center, Houston, TX; ^8^ Weill Medical College at Cornell University, New York, NY

## Abstract

More than 80% of global hepatocellular carcinoma (HCC) patients are estimated to occur in sub-Saharan Africa (SSA) and Eastern Asia. The most common risk factor of HCC in SSA is chronic hepatitis B virus (HBV) infection, with the incidence highest in West Africa. HBV is highly endemic in SSA and is perpetuated by incomplete adherence to birth dose immunization, lack of longitudinal follow-up care, and impaired access to antiviral therapy. HBV may directly cause HCC through somatic genetic alterations or indirectly through altered liver function and liver cirrhosis. Other risk factors of HCC in SSA include aflatoxins and, to a lesser extent, African iron overload. HIV plus HBV co-infection increases the risk of developing HCC and is increasingly becoming more common because of improving the survival of patients with HIV infection. Compared with the rest of the world, patients with HCC in SSA have the lowest survival. This is partly due to the late presentation of HCC with advanced symptomatic disease as a result of underdeveloped surveillance practices. Moreover, access to care and resource limitations further limit outcomes for the patients who receive a diagnosis in SSA. There is a need for multipronged strategies to decrease the incidence of HCC and improve its outcomes in SSA.

## INTRODUCTION

Hepatocellular carcinoma (HCC) is the sixth most common cancer and the fourth most common cause of cancer-related death worldwide.^1^ In 2017, there were an estimated 953,000 incident cases of liver cancer globally and 819,000 deaths because of liver cancer. Liver cancer was more common in men, with one in 42 men developing liver cancer compared with one in 118 women.^2^ The main risk factors of HCC include chronic viral hepatitis B virus (HBV and hepatitis C virus infections), alcohol overuse, and nonalcoholic fatty liver disease (NAFLD), mainly because of morbid obesity and diabetes.^3^ There is heterogeneity in the distribution of these risk factors between low- and middle-income countries and high-income countries.^4^ Early-stage HCC is curable with surgical resection, advanced locoregional treatments such as ablation and chemoembolization, and liver transplant.^5^ Treatment of intermediate- and advanced-stage HCC is largely palliative. Yet, recent improvements in systemic therapy, particularly the use of targeted agents with antiangiogenic properties, have led to meaningful improvements in survival.^6-14^

**CONTEXT**

**Key Objective**
Sub-Saharan Africa (SSA) continues to have 80% of the global hepatocellular carcinoma (HCC) patients. Hepatitis B infection is the main etiology, and HIV increases the risk of developing HCC.
**Knowledge Generated**
Late presentation and poor or inexistent surveillance for HCC render survival among patients with HCC in SSA the lowest compared with the rest of the world. Moreover, access to care and resource limitations further limit outcomes for the patients who receive a diagnosis in SSA, and most of the patients do not undergo standard treatment for HCC. This is contributed by the inadequate infrastructure, personnel, and lack of access to health care within many rural communities. By the time a definitive diagnosis has been made, most patients would be of poor performance status and may have unfavorable prognostic markers.
**Relevance**
Thus, comprehensive multipronged strategies to decrease the incidence and improve detection and the treatment of HCC in SSA are needed.

## INCIDENCE AND MORTALITY OF LIVER CANCER IN SUB-SAHARAN AFRICA

**FIG 1:**
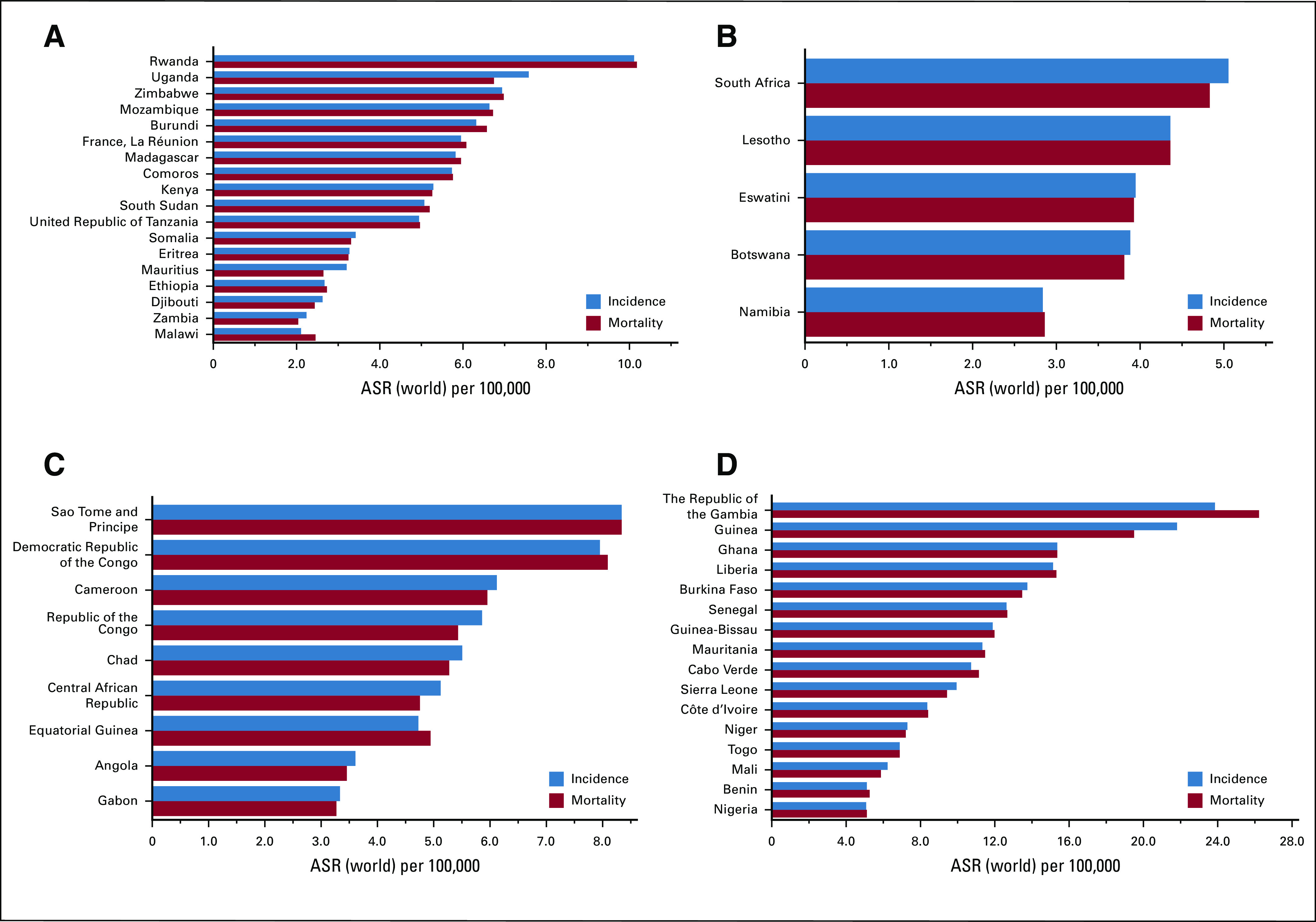
Age-standardized incidence and mortality rates of liver cancer in African countries across different regions of sub-Saharan Africa. (A) Eastern Africa, (B) Southern Africa, (C) Central Africa, and (D) Western Africa. ASR, age-standardized rates (incidence and mortality). Reproduced from GLOBOCAN 2018.^17^

More than 80% of global HCC patients are estimated to occur in sub-Saharan Africa (SSA) and Eastern Asia.^15,16^ For reference, SSA is geographically composed of four regions: Central, West, East, and Southern Africa. The regional age-standardized incidence rates of liver cancer (for all ages, standardized to the world population) are 8.3, 6.5, 4.9, and 4.8 per 100,000 person-years in Western, Central, Southern and Eastern Africa, respectively.^17^ Incidence of HCC in SSA is highest in West Africa, with highest incidence in Gambia and Guinea (Fig 1 and Table 1).^17,18^ The trends in liver cancer incidence over time have been studied only in individual cohort studies and hence may not suggest universal trends in SSA. A study from Uganda and Gambia showed that the incidence of HCC has been increasing over time until mid-to-late 2000s.^19^ Mortality closely parallels the incidence rate in SSA because of the high case fatality rate (Fig 1). In South Africa, from 1999 to 2015, the overall mortality attributable to liver cancer significantly decreased in men (−4.9%) and women (−2.7%).^20^ The same study also showed a racial disparity in mortality, with White South Africans having significantly lower mortality than their Black counterparts.^20^ The incidence and mortality rates are likely underestimated in these efforts as there are multiple hindrances to data collection efforts, such as inadequately trained personnel, inadequate funding and infrastructure, poor access to health care and diagnosis, inadequate population surveys, or unreliable vital statistics.^20,21^

**TABLE 1:**
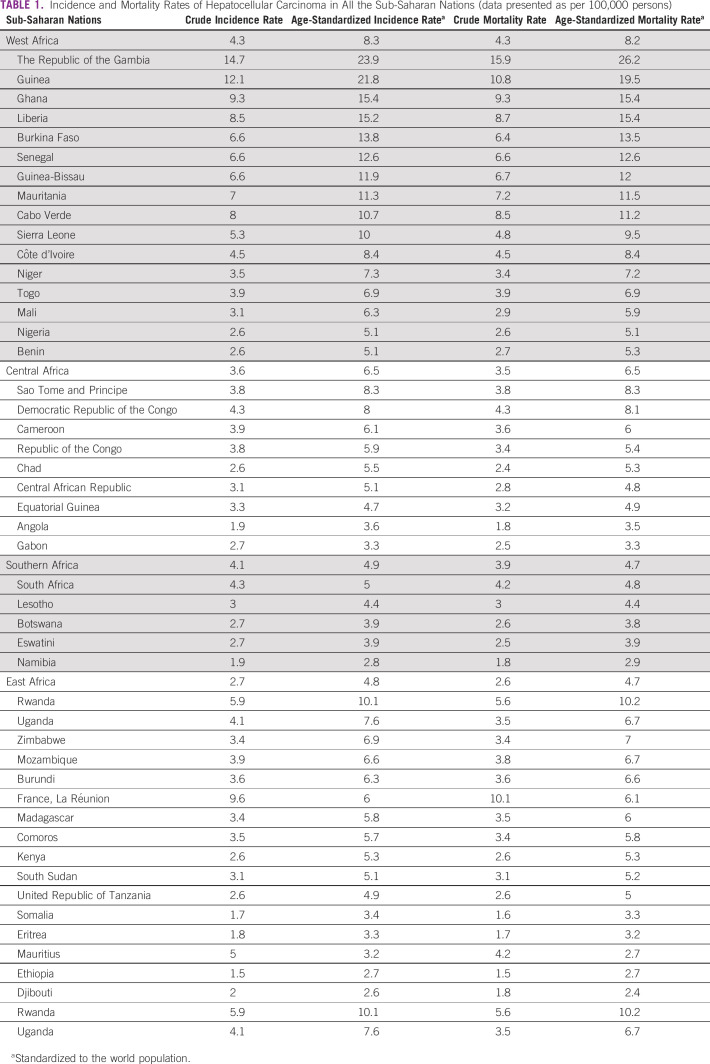
Incidence and Mortality Rates of Hepatocellular Carcinoma in All the Sub-Saharan Nations (data presented as per 100,000 persons)

## RISK FACTORS OF HCC IN SSA

The predominant risk factor of HCC in SSA is chronic HBV infection.^22^ In SSA, HCC affects men more than women; although the risk increases with age, patients in SSA tend to be younger than those in other parts of the world.^21^ The higher level of androgens in men is linked to an increased risk of HBV-induced HCC.^23^ HCC is seen at an earlier age group in SSA because of genetic factors, HBV infection during childhood or birth, impact of prevailing HBV genotypes (and their genetic variants), and aflatoxin exposure.^24,25^ Rural regions have a higher incidence of HCC than urban areas, presumably because of high levels of HBV infection seen in rural areas.^26^ We will review multiple reasons for the high prevalence of chronic HBV infection in Africa in later sections of this review. In addition to viral hepatitis, there are other unique risk factors for HCC in the region.^18^ These include frequent exposure to aflatoxin via grain-based diets prevalent in the region and African dietary iron overload.^27^ The interplay between these additional risk factors and a high burden of HIV in the background of co-infection with HBV may in part explain the high incidence of HCC in this region.

### HBV

**FIG 2:**
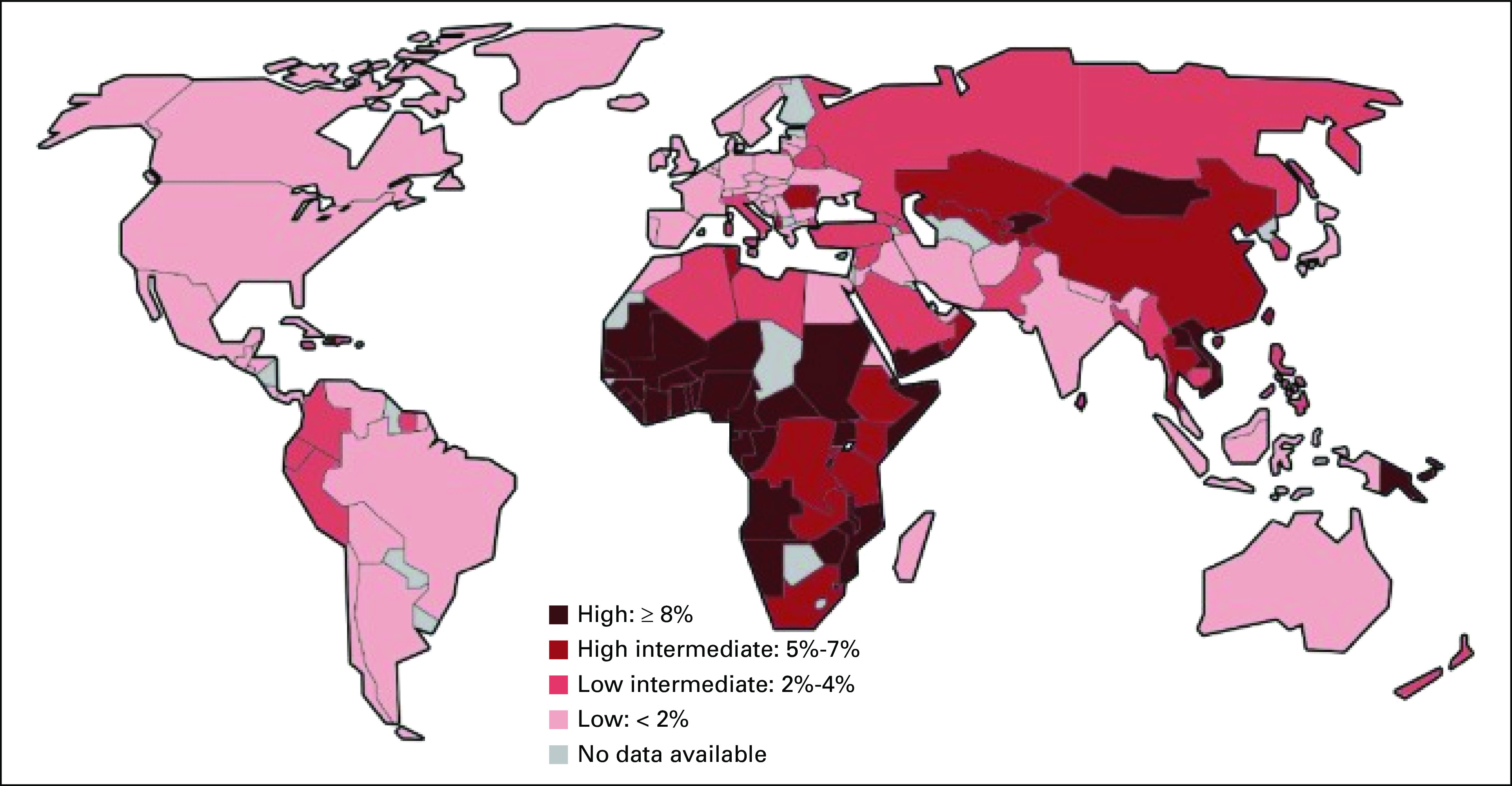
World map depicting the country-wise prevalence of chronic hepatitis B infection. Materials developed by CDC. Reproduced from Schweitzer et al.^30^

Chronic HBV is a global health problem, and its global prevalence in the general population was 3.5% (about 257 million persons), as per the WHO 2017 global hepatitis report.^28^ Viral hepatitis was responsible for 887,000 deaths in 2015, and mortality from viral hepatitis increased by 22% between 2000 and 2015.^28^ Western Pacific has the highest prevalence of chronic hepatitis B infection but has decreased the infection in the upcoming generation of children to < 1% as a result of multiple successfully implemented strategies targeted at birth immunization, mass seroprevalence assays, regional verification of viral control, and inhibiting mother-to-child transmission of chronic hepatitis B infection.^29^ Africa is second to the Western Pacific WHO region in terms of the prevalence of chronic HBV infection (6.1%, 60 million *v* 6.2%, 115 million).^28^ It is hyperendemic (> 8% prevalence of chronic carriers^30^) in some sub-Saharan countries like Nigeria, Gabon, Cameroon, and Burkina Faso; endemic to an intermediate degree (2%-7.99% prevalence)^30^ in Kenya, Zambia, Ivory Coast, Liberia, Sierra Leone, and Senegal; and comparatively endemic to low degree (< 2% prevalence)^27^ in North African countries, such as Egypt, Tunisia, Algeria, and Morocco^30^ (Fig 2). In SSA, HBV transmission occurs predominantly through horizontal route among individuals of the same generation in early childhood.^30^ Primary infection occurs mostly through percutaneous infection from saliva, blood, unsterile needles, unsafe blood transfusions, and tribal scarification (practice of scratching, etching, burning or branding or superficially cutting patterns, or images into skin as permanent body modification^31,32^), among other vehicles.^33^ Birth dose and childhood immunization against HBV in Africa were attempted successfully in certain countries. Several studies from Africa showed a decrease in the prevalence of chronic HBV infection after the introduction of hepatitis B immunization in routine childhood immunization schedules.^34^ However, only 19% of 47 African countries introduced birth dose vaccination by July 2017 despite WHO recommending universal HBV birth dose vaccination by 2005.^34^ There are numerous challenges associated with effective and timely immunization in African countries, such as lack of infrastructure to maintain vaccines in target temperature range from manufacture to recipient (cold chain management), inadequate access to health care in rural areas, high prevalence of home births, inadequacy of national health policies, and cultural practices of inhabitants, among others.^34^

**FIG 3:**
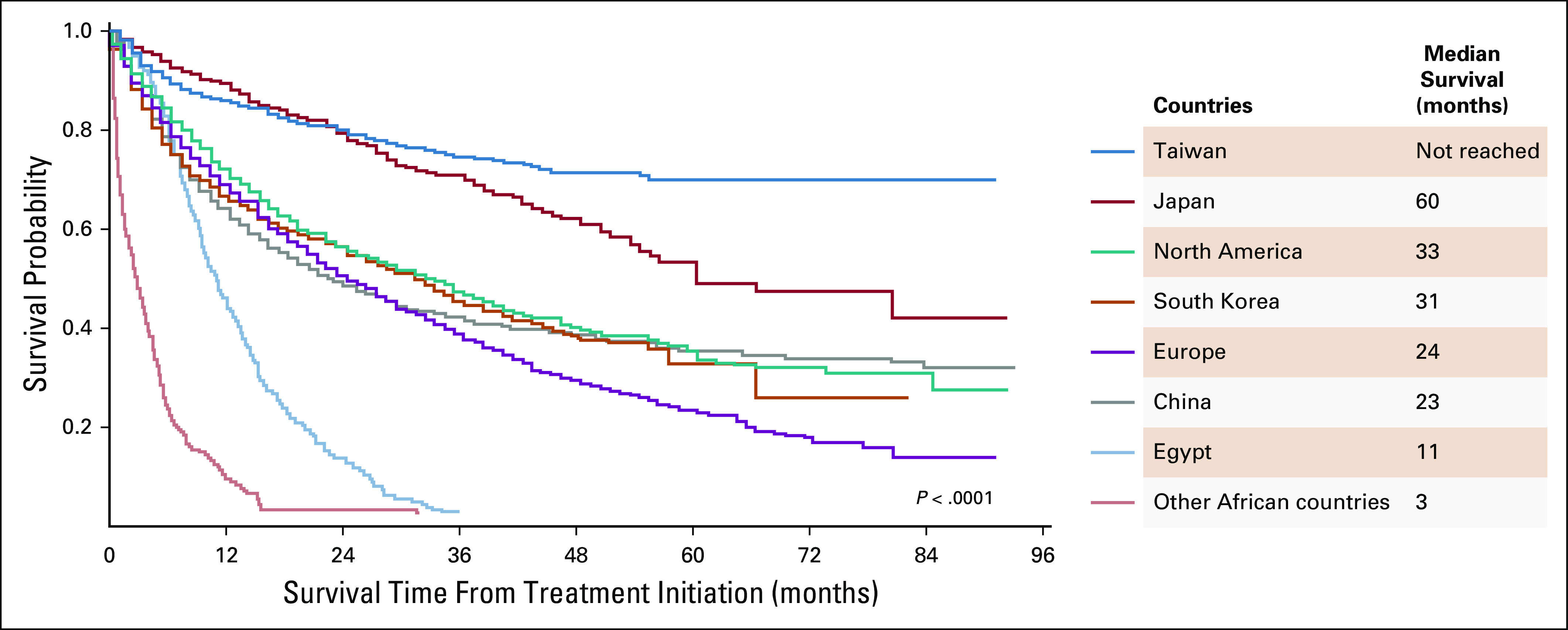
Survival curves of hepatocellular carcinoma in different countries or continents. Reproduced with permission from Yang et al.^15^

Vertical transmission of HBV (from mother-to-child) is a less common means of transmission in SSA compared with the Asia-Pacific region, where it is the predominant transmission route.^35^ A recent systematic review and meta-analysis confirmed that antiviral treatment of pregnant women combined with birth dose immunization and immunoglobulin administration at the time of birth reduces risk of mother-to-child transmission.^36^ However, this has not been recommended in SSA by the WHO or African Health ministries because of the low perinatal transmission rate and lack of clinical trials in the continent.^37^ Currently, birth dose immunization alone is the standard recommended by WHO for African countries for decreasing mother-to-child transmission, but this occurs only in < 3% of infants born in Gambia despite Gambia being one of the first countries in SSA to have implemented national HBV immunization.^37^

WHO estimated that only 10.5% of all patients worldwide with HBV infection knew of their diagnosis as of 2016^28^ and only 5% of those eligible for treatment had received antiviral treatment.^38^ Effective treatment of chronic HBV infection requires surveillance and screening practices, longitudinal care, periodic blood investigations, access to health care, and affordability of medications. In resource-limited SSA, this is a major hurdle.^39^ In SSA, < 1% of HBV-infected individuals were aware of their diagnosis despite the availability of reliable testing modalities and up to 15% entered care with liver cirrhosis.^40^ Tenofovir, WHO-preferred treatment for chronic hepatitis B, is available for purchase from the private sector hospitals and pharmacies, but most patients cannot afford its lifelong supply as they have to pay out of pocket in private sector.^41^ Public sector hospitals serve most of the African population who cannot afford care in the private sector. Although tenofovir is available as part of antiretroviral therapy and is accessible to all through the public sector hospitals, it is not accessible to monoinfected hepatitis B patients.^42^ Nationally funded actional plans in the public sector have been adopted by some African nations, but published experiences of such executions are limited to the PROLIFICA study in Gambia and another pilot program in Ethiopia.^41,43^ These are examples of large screen-and-treat operations at a national level that demonstrate virologic control can be achieved with mass screening and prompt linkage to care and easy access to medications.

The combined ineffectiveness of providing universal immunization to newborns in Africa and the major hurdles involved in the treatment of patients with chronic hepatitis B infection are the main reasons behind the majority of them being at risk for development of the HCC, a late sequela of the infection.^34^

Chronic HBV infection leads to liver cirrhosis over time, and its risk is higher in those with positive HBeAg, an elevated HBV DNA level, and HBV genotype C.^44^ HCC occurs in HBV-related cirrhosis at an annual rate of 2.3%. Accumulation of mutations in numerous driver genes, p53-RB pathway, β-catenin or WNT pathway, phosphatidylinositol 3-kinase-mammalian target of rapamycin pathway, and NRF2-KEAP1 pathway of hepatocytes is seen in HCC developing from cirrhosis.^45^ Chronic HBV infection can also directly lead to HCC.^46^ A high number of HBV-DNA integrations are randomly noted in the chromosomes of HBV-infected livers.^47^ HBV genome integrates into the TERT gene in high clonal proportion.^48^ HBx gene of the HBV genome likely interferes with telomerase activity during hepatocyte proliferation.^47^ HBV also affects epigenetic mechanisms, and the viral genome-encoded proteins have been associated with malignant potential.^46^ The protein encoded by the Hbx gene, HBx protein, is a transactivating factor. It transactivates binding sites for AP-1 and NF-κB, activates p53-RB and β-catenin pathways, and is also involved in transcriptional modulation.^46^ Occult chronic HBV infection (persistent viremia in the context of negative HBsAg and positive HBcAg) also leads to HCC carcinogenesis.^49^ This could mean that we are underestimating the proportion of HCCs attributable to HBV, as most studies do not include checking HBV DNA levels to ascertain etiology.^37^

In SSA, HBV genotype E predominates and is seen through the region (especially West and Central Africa).^50^ This is followed by genotype A, which is the predominant genotype in Uganda and Cameroon. Both these genotypes have a high oncogenic potential compared with the others.^27^

Perinatal vertical transmission of HBV is more likely to occur if the mother is HBeAg-positive and has a high viral load.^51^ Although HBeAg clears at an early age in Africans, younger African mothers have a higher risk of vertical transmitting HBV infection.^52^ HCC patients are more commonly seen with first birth order in the family, a marker of younger maternal age,^52^ and could be a result of the interaction between the phenomena that older age is protective of vertical transmission and the fact that vertically acquired HBV infection has a higher likelihood of progression to HCC.^53^

### HIV and HCC

Africa is the epicenter of the AIDS epidemic and accounts for up to 71% of AIDS patients worldwide.^54^ Antiretroviral therapy has changed a once highly fatal disease entity into a chronic illness with markedly improved survival. This has let other comorbidities such as liver disease to become a major concern, which once did not have a chance to manifest in HIV-infected individuals.^27^ Co-infection with HIV and HBV is common because of shared means of transmission, and there are close to around 2-4 million patients.^55^ The mean proportion of HIV patients with HIV-HBV co-infection in SSA is 7.8% and varies from 0% to 28.4% in different regions of SSA.^52^ The co-infection rates among HIV-positive children and pregnant women were 3.8% and 7.4%. These rates are based on too few population-based surveillance programs and mostly arise from observational cohort studies. West African countries seem to have the highest co-infection rates, followed by Southern African countries. The least co-infection rate is seen in East Africa. Studies in patients with HIV of South Africa and West Africa reported occult infections (as evidenced by positive HBV DNA and negative HBsAg) to be around 10%-33%. Multiple studies in the United States and Europe have shown that HIV co-infection with HBV enhances the hepatocarcinogenic potential.^56^ The immune T-cell response against HBV is impaired in HIV-HBV–co-infected patients leading to higher levels of HBV replication, decreased rates of resolution of HBV infection, and increased rates of HBV reactivation.^57^ HIV infection by itself also increases reactive oxygen, activates hepatic stellate cells, and promotes immune-mediated injury by Kupffer cells in the liver. HIV infection also causes an increased translocation of gut bacteria, leading to increased lipopolysaccharide-induced toll-like receptor activation. HIV medications also have a hepatotoxic effect leading to increased risk of HCC.^53^ Certain mutations of HBV genome, such as PreS deletion, are associated with increased risk of HCC in HBV monoinfected individuals and are also found more commonly in the HBV-HIV–co-infected individuals.^58^ However, cohort studies of co-infected individuals with HBV genome mutations are required to study the risk of HCC they confer. A study from Africa revealed that HIV-HBV–co-infected individuals did not have an increased incidence of HCC, but this was done at a time when the survival for patients with HIV was quite poor.^59^ However, systematic cohort studies evaluating the natural history of HBV-HIV–co-infected individuals in Africa are still lacking.

### Aflatoxins

Aflatoxins derived from *Aspergillus flavus* and *Aspergillus parasiticus* are carcinogens and are synergistic with HBV infection in causing HCC.^60^ They are also partly responsible for young age of onset of HBV-associated HCC in SSA.^61^ Metabolites of aflatoxins intercalate into the host genome, leading to specific gene mutations, particularly of the p53 tumor suppressor gene.^62^ The population attributable risk of aflatoxins is 21% in chronic HBV carriers and 8.8% in HBsAg negative populations.^63^ The warm and humid climate coupled with the staple diet (consisting of maize and rice) that is sensitive to aflatoxin production and improper postharvest storage practices is the reason behind the high prevalence of aflatoxin contamination of foods in SSA.^64^ High aflatoxin concentrations were found in foods such as nuts in Nigeria, rice grains in Ghana, peanuts in Zambia, locally brewed alcoholic drinks in Kenya and Ethiopia, and cow milk in Cameroon.^27^ Aflatoxin exposure will only become worse with global warming, and there is a general lack of awareness about this issue among the general public of Africa.^64^ There is a lack of effective interventions that have decreased this exposure systematically.

### African Dietary Iron Overload

Rural dwellers in Africa (mainly in Nigeria) consume large quantities of home-brewed beer, which are made by fermenting locally produced crops in large iron or steel containers.^27^ The resultant beer contains a high concentration of bioavailable iron leached from iron and steel ware.^65^ This leads to an accumulation of iron in livers of certain African individuals with functional differences in ferroportin, a protein for iron transport found in hepatocytes.^66^ The iron accumulation in the liver is a risk factor for the development of HCC irrespective of liver disease.^67^ It is termed African dietary iron overload.

### NAFLD

According to the WHO, obesity is an emerging problem of the developing world, with its associated comorbidities on the rise in Africa.^68^ The main risk factors are morbid obesity and type 2 diabetes, which are magnified by rapid urbanization, physical inactivity, nutrition transitions, socioeconomic changes, and aging.^69,70^ In Africa, the combination of obesity, type 2 diabetes, and NAFLD is associated with the late presentation of larger tumors not amenable to curative therapy.^71^ Several studies have demonstrated that once NAFLD sets in, Africans are at similar risk of progression to cirrhosis and HCC as Whites.^72,73^

## MANAGEMENT OF HCC AND ACCESS TO CARE IN SSA

Patients generally first present in a district hospital setting where they would be attended to in general medical or internal medicine facilities, before referral to the tertiary centers. Unfortunately, by the time a definitive diagnosis has been made, most patients would be of poor performance status and may have unfavorable prognostic markers.

HCC in SSA has significantly poorer median survival compared with Egypt and all other countries (Fig 3). Most patients in SSA tend to present with symptoms to a district hospital where they would be attended by internal medicine services and eventually referred to tertiary centers. In one of the most comprehensive international multicenter retrospective studies comparing HCC outcomes in Egypt and SSA, patients in SSA were diagnosed with more severe liver dysfunction (Child-Pugh scores B and C 64% *v* 93%), worse Eastern Cooperative Oncology Group performance status (Eastern Cooperative Oncology Group 2-4 15% *v* 67%), and advanced Barcelona-Clinic Liver Cancer (BCLC) stage (BCLC stage D 7% *v* 72%).^22^ This is likely a reflection of the lack of HCC surveillance programs, the absence of trust in health systems, and poor health-seeking behavior in SSA.^74,75^ Only 3% of the patients with HCC received any disease-specific treatment in tertiary care centers of SSA (Table 2).^22^ None of them underwent liver transplantation, and sorafenib was used only in 1% of them despite 23% presenting in BCLC stage C.^22^ Tertiary referral centers can provide care to select few in SSA who can afford it financially, and hence, overall treatment rate for patients with HCC is probably much lower.^22^ Anecdotally, the care providers in SSA are of varying cadres, ranging from clinical officers (United States equivalent of advanced practice provider), medical officers who have trained in a medical school and internship but are nonspecialized, to consultants with specialization in surgery, internal medicine, and other specialties.

**TABLE 2:**
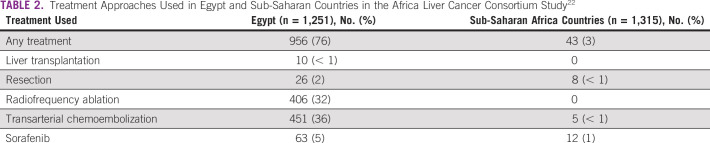
Treatment Approaches Used in Egypt and Sub-Saharan Countries in the Africa Liver Cancer Consortium Study^22^

There are 102 establishments in all of Africa that provide treatment for patients with cancer, 38 of which are in South Africa.^76^ Close to 22 African nations do not have palliative care facilities. An estimated 88% of all patients who report moderate to severe pain eventually die without receiving any pain treatment.^77^ Lack of provider training and unreasonable fears of patients toward opiates contribute to this trend.^77^ Liver transplantation in SSA is reported to be performed at two centers in South Africa, and it is practiced in a limited number of private health care facilities.^78^ There are less than two surgeons per 100,000 inhabitants in SSA (excluding South Africa), in comparison with 35 surgeons in England per 100,000 inhabitants,^76^ and oncologic surgery is mainly performed by general surgeons in SSA.^79,80^ East, West, and Central Africa had the highest unmet need for surgical care.^81^

In a cross-sectional study of a cancer center in Tanzania, only 50% of cancer-directed systemic therapies were available and more than 70% of patients did not receive adequate therapy.^82^ Evidence-based systemic therapy is generally available at a great cost and is affordable to a minority of patients in SSA. Cancer drugs in SSA cost close to 4 months of average income, and as most patients are not insured, only a few can afford them.^74^ Less than 1% of HCC clinical trials take place in Africa.^83^ A survey of radiology training programs in SSA does not mention training in interventional radiology, and there are too few general radiologists in comparison with the patient population.^84^ Despite the advent of experimental use of radiation therapy for HCC, 3D conformal radiotherapy is scarcely available in SSA and usually dedicated to potentially curable cancers as an essential component, such as cervical and breast cancers.

## FUTURE DIRECTIONS

Measures for primary prevention of HBV infection, such as HBV birth vaccination and follow-up programs, measures to prevent maternal-to-child transmission of HBV, and screening of pregnant women for HBsAg have been started in many regions of SSA.^85^ These should be pursued with vigor to achieve 100% coverage. Delivery of universally accessible antiviral treatments, screening programs for HCC, and reduction of dietary aflatoxin exposure can markedly reduce HCC incidence and improve mortality. The African Cancer Registry Network (AFCRN) provides expert evaluation of current challenges and technical support to address the identified barriers.^86^ The long-term goal of AFCRN is to strengthen health systems and create research platforms for problem identification. The AIDS Malignancy Consortium is a National Cancer Institute (NCI)–supported clinical trials group with a strong presence in Africa, helping investigating new treatment and prevention of interventions and studying the pathobiology of malignancies in people living with HIV both in the United States and internationally, has a strong interest in helping patients with HCC and HIV.^84^ Novel guidelines for the management and treatment of HCC in SSA, which would be dependent on the resources available, are badly needed. Such effort led by most authors plus other colleagues in SSA is already underway. Finally, the development of biomarkers and new therapeutic interventions will need a better understanding of the unique genetic and epigenetic characteristics of HCC in SSA. Clinical and genetic research collaborations centered on African populations are required for achieving this.

In conclusion, HCC is very common in SSA because of the endemicity of chronic HBV infection. Other risk factors that are unique to SSA are rural background, aflatoxin exposure, and dietary African iron overload. There are multiple methods to decrease the incidence and transmission of HBV, which have been inadequately used in SSA. In SSA, HBV infection is the most common etiologic cause HCC, and this risk is increased with HIV co-infection. Compared with higher-income countries, patients with HCC living in SSA have among the poorest reported survival. This is due to the late presentation of these tumors in the symptomatic phase and inadequate surveillance practices. Most of the patients do not undergo standard treatment for HCC in SSA. This is due to inadequate infrastructure, personnel, and lack of access to health care within many rural communities. Comprehensive multipronged strategies are required to decrease the incidence and improve detection and the treatment of HCC in SSA.
